# Unlocking Potential: Low Bovine Serum Albumin Enhances the Chondrogenicity of Human Adipose-Derived Stromal Cells in Pellet Cultures

**DOI:** 10.3390/biom14040413

**Published:** 2024-03-28

**Authors:** Isabel Casado-Losada, Melanie Acosta, Barbara Schädl, Eleni Priglinger, Susanne Wolbank, Sylvia Nürnberger

**Affiliations:** 1 Department of Orthopedics and Trauma-Surgery, Division of Trauma-Surgery, Medical University of Vienna, 1090 Vienna, Austria; isabel.casadolosada@meduniwien.ac.at (I.C.-L.); melanie.acosta@meduniwien.ac.at (M.A.); 2 Ludwig Boltzmann Institute for Traumatology, The Research Center in Cooperation with AUVA, 1200 Vienna, Austriaeleni.priglinger@jku.at (E.P.); susanne.wolbank@trauma.lbg.ac.at (S.W.); 3 Austrian Cluster for Tissue Regeneration, 1200 Vienna, Austria; 4 University Clinic of Dentistry, Medical University of Vienna, 1090 Vienna, Austria; 5 Department for Orthopedics and Traumatology, Kepler University Hospital GmbH, Johannes Kepler University Linz, 4020 Linz, Austria

**Keywords:** bovine serum albumin, adipose-derived stromal/stem cells, chondrogenic differentiation, hypertrophy

## Abstract

Bovine serum albumin (BSA) plays a crucial role in cell culture media, influencing cellular processes such as proliferation and differentiation. Although it is commonly included in chondrogenic differentiation media, its specific function remains unclear. This study explores the effect of different BSA concentrations on the chondrogenic differentiation of human adipose-derived stromal/stem cells (hASCs). hASC pellets from six donors were cultured under chondrogenic conditions with three BSA concentrations. Surprisingly, a lower BSA concentration led to enhanced chondrogenesis. The degree of this effect was donor-dependent, classifying them into two groups: (1) high responders, forming at least 35% larger, differentiated pellets with low BSA in comparison to high BSA; (2) low responders, which benefitted only slightly from low BSA doses with a decrease in pellet size and marginal differentiation, indicative of low intrinsic differentiation potential. In all cases, increased chondrogenesis was accompanied by hypertrophy under low BSA concentrations. To the best of our knowledge, this is the first study showing improved chondrogenicity and the tendency for hypertrophy with low BSA concentration compared to standard levels. Once the tendency for hypertrophy is understood, the determination of BSA concentration might be used to tune hASC chondrogenic or osteogenic differentiation.

## 1. Introduction

Albumin is the most abundant protein present in blood plasma, comprising around 60% of the total protein content. It is a small protein of 66 kDa with 585 amino acid residues, synthesized in polysomes bound to the endoplasmic reticulum of hepatocytes in the liver at a range of 3.5–5.5 g/dL [1]. Albumin is released into the blood, but it transits from the intravascular to the extravascular space several times during the day. In total, it predominantly resides in the extravascular compartment, with 40% in plasma and 60% in the extravascular space [2,3,4,5].

Due to its strong net negative charge, its main role, physiologically, is to modulate the colloidal osmotic and oncotic pressure of plasma, thereby regulating fluid distribution. Hence, albumin is also known for its transport and binding/ligand capacity to water, fatty acids, hormones, bilirubin, metal ions, as well as drugs, metabolites, and/or amino acids [2,6,7]. The binding properties of Human Serum Albumin (HSA) depend on its three-dimensional structure, which comprises three homologous domains and many α-helices, forming a heart-shaped tertiary structure. Beyond its primary functions, albumin exhibits antioxidant effects, safeguarding against lipid peroxidation induced by inorganic reactive oxygen species (ROS). It also possesses anti-inflammatory properties, regulating interactions between neutrophils and endothelial cells [8,9].

Albumin is used in the clinical routine as a supplement to blood for the maintenance and restoration of blood volume after hemorrhagic shock, or in clinical situations such as hypoproteinemia, hypovolemia, and fetal erythroblastosis [10,11]. These things considered, albumin has been used in mammalian cell culture practices as one of the main constituents of Fetal Bovine Serum (FBS) [also known as Fetal Calf Serum (FCS)], comprising 95% of the total protein content (~5 mg/mL) [12]. Since FBS was discovered to promote cellular growth in cell and tissue cultures in the late 1950s by T. Puck [13], it has been widely used for cell cultures. FBS was shown to promote cell attachment, growth, and proliferation for a vast number of cell types. However, there are several concerns regarding the use of FBS, which have promoted the search for alternatives. These include (1) ethical concerns related to its collection from unborn fetal calves at the slaughterhouse without humane and pain management procedures; (2) intra- and inter-laboratory reproducibility concerns because of the unknown composition, variation between brands, and geographical batch-to-batch variation; (3) proneness to contaminations from prion proteins, endotoxins, microbes, or viruses; and (4) possible interference with phenotypic cell stability, as well as the expression of non-human N-glycolylneuraminic acid (Neu5Gc), which has been found to influence tumor progression [14]. Over the past decade, an increasing number of researchers have shifted towards employing FBS-free or xeno-free (XF) media. The adoption of these alternatives is deemed to be crucial for the effective translation of various cell types or therapies into clinical practice. Noteworthy alternatives include human serum, human platelet lysate (hPL), sericin protein, earthworm heat-inactivated coelomic fluid (HI-CF), and chemically defined serum-free (SF) and xeno-free (XF) media [15,16]. Albumin has also emerged as an alternative to FBS and is often incorporated in SF and XF media since it can be of natural origin, isolated from humans or animals, or synthetic. Several authors have compared the use of FBS with both human and bovine serum albumin and shown improved cell proliferation and growth for several cell lines like C2C12, plus the maintenance of multipotency of mesenchymal stromal cells (MSCs) such as hASCs [17,18,19].

At a cellular level, albumin influences several cellular processes given its binding capacity. Hence, it acts as the main carrier of fatty acids (FA) and is responsible for the transfer of cholesterol, among others, between fibroblasts and lipoproteins. These things considered, different studies suggest that HSA interacts directly with FA and FA-binding proteins of the plasma membrane, influencing cell survival, apoptosis, or stem-cell fate [20]. As part of the cell culture medium, BSA has also been shown to modify the differentiation phenotype of MSCs. MSCs, especially bone-marrow-derived stromal cells (BM-MSCs) and ASCs are the main alternative cell type to the use of articular chondrocytes (ACs) for articular cartilage regeneration. Therefore, choosing the appropriate culture conditions to elicit this phenotype is critical for translation into clinical routine. For chondrogenic differentiation of both ASCs and articular chondrocytes from different species, different XF and SF culture media are employed. These media are often supplemented with BSA or HSA at concentrations ranging from 0.5 to 1.25 mg/mL, with the last one being more frequently used [15,21,22,23]. hPL has also been extensively investigated as an alternative to FBS for chondrocyte maintenance and chondrogenic differentiation with intricate results. With a lower protein and albumin content, hPL has been found to promote chondrogenic differentiation of human cartilage-derived progenitor cells compared to the FBS-containing medium [24]. Furthermore, commercial HSAs, such as the low molecular weight fraction of 5% HSA (LMWF-5A), have been found to prime BM-MSCs into the chondrogenic lineage, as well as to re-differentiate fibroblast-like osteoarthritic chondrocytes, with increased production of collagen type II [25,26]. Despite albumin being supplemented both in the differentiation and expansion culture medium, the role and mechanisms of action remain to be elucidated [27,28,29,30]. Additionally, the adequate concentration of BSA or HSA for chondrogenic differentiation has not been sufficiently investigated. Hence, the purpose of this study was to elucidate the effect of BSA concentration on the chondrogenic differentiation of primary hASCs via 3D pellet cultures. To do so, pellet size measurement, biochemical analysis, including glycosaminoglycans (GAG) content, and gene expression were analyzed. Additionally, histology was used to investigate chondrogenic differentiation. Our observations will provide insights into the influence of BSA in the chondrogenic differentiation process, which should allow us to define appropriate chondrogenic media for hASCs, leading us a step closer to developing a xeno-free and serum-free formulation. Second, this study will enlighten some of the improvements in the culture conditions of MSCs. To our knowledge, this is the first study investigating differential concentrations of BSA on the chondrogenic differentiation of hASCs.

## 2. Materials and Methods

### 2.1. Cells and Media

Human adipose-derived stromal cells (hASCs) were harvested and isolated at the Red Cross Blood Transfusion Service of Upper Austria. They were isolated from human fat tissue obtained from routine liposuctions as previously described [31]. Subcutaneous adipose tissue was obtained from 6 female donors [age: 43 ± 8 years; body mass index (BMI): 27.93 ± 8.53 kg/m^2^], with one of them being obese (BMI: 43.5 kg/m^2^). Briefly, enzymatic digestion was performed with collagenase (1.5 mg/mL collagenase I, 20 mg/mL BSA) and 25 mM N-2-hydroxy ethylpiperazine-N′-2-ethanesulfonic acid in PBS under vigorous shaking for 60 min at 37 °C.

Cells were expanded in Endothelial Cell Growth Basal Medium (EGM-2, Lonza, Vienna, Austria CC-3156 and CC-4176), based on previous studies [32,33]. hASCs in passage 2 were differentiated towards the chondrogenic lineage using different BSA concentrations. The chondrogenic differentiation medium was composed of DMEM high glucose (Sigma-Aldrich, Merck KGaA, Darmstadt, Germany D6546), supplemented with ITS 100× premix (Gibco, Fisher Scientific, Schwerte, Germany), 0.17 mM L-ascorbic acid 2-phosphate (Sigma), 1 mM sodium pyruvate (Gibco), 0.35 mM L-proline (Sigma), 100 U/mL Penicillin/Streptomycin (Sigma), 2 mM L-Glutamine (Gibco), and it was freshly supplemented with 20 nM dexamethasone (Sigma), 10 ng/mL rhBMP-6 (R&D, Minneapolis, MN, USA 507-BP), and 10 ng/mL TGF-β3 (R&D systems, 8420-B3). Besides, BSA (Sigma, A9647-50G) was added to the media resulting in the following concentrations: (1) high dose (HD) 1.25 mg/mL, (2) middle dose (MD) 0.125 mg/mL, and (3) low dose (LD) 0.0125 mg/mL BSA.

### 2.2. Pellet Formation

Before the pellet culture, 96-well U-bottom plates (CellStar; Greiner, Kremsmünster, Austria) were coated with poly (2-hydroxyethyl methacrylate) (poly HEMA, Sigma). For this, 0.5 g poly-HEMA was dissolved in 95% ethanol on a shaker overnight at 38 °C. Afterwards, 50 µL of the solution was added to each well, and the plates were left at 37 °C for overnight shaking to evaporate ethanol, followed by UV sterilization for at least 2 h. For each donor, the three previously mentioned BSA concentrations were used, with 50–60 pellets for each concentration, except for donor 3 where the limited number of cells only allowed for the preparation of 25 pellets. Pellets were created by seeding 200,000 cells/pellet in a total volume of 200 μL and centrifuging at 300× *g* for 5 min. They were cultured in a medium containing 10 ng/mL TGF-β3 and BMP-6 combined with each BSA concentration, which was replaced twice a week with a partial medium exchange (170 μL) for the whole differentiation time of five weeks. Before seeding, 1 × 10^6^ cells were collected for normalization of qRT-PCR (d0). These things considered, for qRT-PCR, 12 pellets (i.e., 4 pooled pellets with *n* = 3 replicates) were harvested at the endpoint of the experiment. Similarly, for GAG/DNA analysis (*n* = 12 pellets), 3 replicates of 4 pooled pellets were harvested at the endpoint of the experiment (d35). For immunohistochemistry, 3 pellets per donor and condition were processed.

### 2.3. Pellet Size Measurement

For size observation, brightfield pictures were taken with a Nikon (Tokyo, Japan) Eclipse TE200-U microscope and NIS-Elements BR 4.20.03 software at 4× magnification every week (days 7, 14, 21, 28, and 35). All pellets of each donor were measured (25–60 per donor) with custom-made software. The field size could be adjusted to the shape of the pellet. Data were saved as an Excel file and analyzed with GraphPad Prism 9.4.0 (La Jolla, CA, USA).

### 2.4. (Immuno-)histochemistry

Randomly, three pellets (*n* = 3) were chosen from each donor and the BSA concentration at d35 and fixed in 4% neutral buffered formalin (Roti^®^ Histofix, Carl Roth, Karlsruhe, Germany, P087.5) for 3 h on a shaker at RT (room temperature). Subsequently, samples were rinsed with DPBS (Gibco, 14190-094) for 10–15 min at RT, followed by dehydration with a graded series of ethanol, and afterwards, xylene (Carl Roth, Karlsruhe, Germany, 9713.4) was added before paraffin embedding. A rotary microtome (Fisher Scientific, Thermo Fisher, Schwerte, Germany, MICROM HM355S) was used to cut 4 µm thin sections. Following deparaffinization and rehydration with a graded ethanol series and distilled water, Safranin O (Saf O) staining was conducted as follows: the sections were immersed in Weigert’s Iron Hematoxylin for 5 min, followed by thorough rinsing and washing in distilled water. Subsequently, the sections underwent additional staining in a 0.02% fast green solution for 1 min, followed by incubation in 1% acetic acid for 30 s and in a 1% Safranin O solution for 30 min. Post-staining, the sections were rinsed in 95% and 100% ethanol, followed by xylene, and ultimately mounted with Consul Mount (Fisher Scientific, ThermoFisher, Schwerte, Germany 12658016). Alcian blue (0.1% in 3% acetic acid at pH 2.5) with Mayer’s Hämalaun counterstaining was used to detect GAGs. To visualize cell distribution and morphology, sections were subjected to Heidenhain’s Azan Trichrome (short “Azan” for azocarmine and aniline blue) displaying the nuclei in bright red. For collagen type II immunostaining, antigens were retrieved using pepsin treatment (pH 2), and samples were incubated with collagen type II primary antibody (Thermo Fisher Scientific, Ms-306-P1-MA5-13026, clone 3B3 1:100) for 1 h at RT. Collagen type I (Thermo Fisher Scientific, PA1-26204, rabbit 1:100), osteocalcin (Merck KGaA, Darmstadt, Germany) AB10911, rabbit 1:200), and collagen type X (Abcam, Cambridge, UK ab 49945, mouse 1:100) primary antibodies were also incubated for 1 h at RT. For MMP-3 (Abcam, ab52915, rabbit 1:50) and MMP-13 (Abcam, ab219620, rabbit 1:50), primary antibodies were incubated overnight. Afterwards, they were incubated with BrightVision Poly-HRP secondary antibody (VWR). For detection, the NovaRED™ peroxidase substrate kit (Vector Laboratories, Szabo Scandic, Vienna, Austria) was used.

### 2.5. Biochemical Assays

At the endpoint of the experiments (day 35), four pellets in three replicates (*n* = 12, 3 × 4 pooled pellets) were randomly selected and digested with a papain solution, following a protocol adapted from Kim et al. [34]. GAG content was quantified using the dimethyl methylene blue (DMMB) assay described by Farndale et al. [35]. Briefly, the DMMB (Sigma) solution was freshly prepared, and the pH was adjusted to 3. A standard dilution series of known concentrations of chondroitin 4-sulfate (Sigma) 1 mg/mL was used to normalize the samples. Thereafter, samples were vortexed and 50 µL was transferred to a clear 96-well flat-bottom well plate (Costar, Boston, MA, USA) with 200 µL of DMMB reagent. DNA content was quantified using the Quant-iT PicoGreen dsDNA Kit (Invitrogen™, Thermo Fisher Scientific, Vienna, Austria) according to the manufacturer’s protocol. Shortly, samples were vortexed, and 1 µL was transferred to a black flat-bottom 96-well plate (Greiner, Kremsmünster, Austria, 655090) in triplicates followed by the addition of 99 µL PicoGreen solution and 4 min incubation, shaking under light exclusion. A standard curve, consisting of known dsDNA concentrations supplied by the manufacturer, was employed to standardize the samples. Then, they were measured in a TECAN Spark™ 10M (Tecan, Gödrig, Austria) plate reader and calculated using linear regression to the standard curve.

### 2.6. mRNA Isolation and qRT-PCR

Pellets were lysed in triplicates (*n* = 12, 3 × 4 pooled pellets) at the endpoint of the experiment (d35) in RLT lysis buffer (RNA lysis buffer from Qiagen, Hilden, Germany 1015750) containing 10 µL/mL β-mercaptoethanol (Sigma, M31148). mRNA was isolated with the RNeasy Micro Kit (Qiagen) according to the manufacturer’s instructions, with 15 uL UltraPure DEPC-treated water (Invitrogen™, Thermo Fisher Scientific, Vienna, Austria) elution. RNA concentrations were measured with a NanoDrop 2000c spectrophotometer (Thermo Fisher Scientific). For reverse transcription, the iScript cDNA synthesis kit (Bio-Rad Laboratories, Vienna, Austria 170-8891) was used on every RNA sample according to the manufacturer’s protocol using a Thermocycler (MWG PRIMUS, MWG Biotech AG, Ebersberg, Germany).

The real-time qPCR was performed using the SensiMix II probe kit (Bioline, LabConsulting, Vienna, Austria, BIO-83005) and TaqMan probes in 20 µL reactions with a 7500 Fast Real-Time PCR system. (Applied Biosystems, ThermoFisher, CA, USA) All primers were purchased from Applied Biosystems, CA, USA with FAM as a dye—*Col2a1* (*Col2*, Eurogentec, Seraing, Belgium, forward: 5′-GCC-TGG-TGTCAT-GGG-TTT-3′, reverse: 5′-GTC-CCT-TCTCAC-CAG-CTT-TG-3′, probe: 5′-AAA-GGT-GCCAAC-GGT-GAG-CCT-3′), *Col1a1* (*Col1*, Applied Biosystems, Hs00164004_m1), *aggrecan* (*ACAN*, Applied BiosystemsHD00153936_m1), *versican* (*VCAN*, Applied Biosystems, Hs00171642_m1), and the housekeeping gene *HPRT1* (Applied Biosystems, Hs02800695_m1). Data were analyzed with the ΔΔCt-method where all the d0 values of the target gene and the mean of the housekeeping gene HTPR1 were used for normalization (ΔCt target − ΔCt HTPR1).

### 2.7. Statistical Analysis

All statistical tests were performed with GraphPad Prism 9.4.0 (La Jolla, CA, USA). To analyze the GAG content and RT-PCR (gene expression of *Collagen II* and *ACAN*), normality could not be tested due to the low *n* number (*n* = 3). Therefore, a one-way Brown–Forsythe and Welch ANOVA test with Dunnet’s correction for multiple comparisons with multiplicity-adjusted *p*-values was performed.

The pellet size distribution was tested for normality with the Shapiro–Wilk test and Q-Q plots. To evaluate the differences between the BSA concentrations (HD, MD, LD) for each independent donor, the endpoint of the experiment (d35) was used, and the relative growth increase was computed. Depending on the distribution, a one-way Brown–Forsythe and Welch analysis of variance (ANOVA) test with Dunnet’s correction for multiple comparisons or Kruskal–Wallis with Dunn’s correction was performed, with a multiplicity-adjusted *p*-value. Groups were considered to be significantly different with an α-value < 5% (*p* < 0.05). Bar charts are given as mean ± standard deviation.

## 3. Results

### 3.1. BSA Influences hASC Pellet Size in a Concentration- and Donor-Dependent Manner

hASC pellets were cultured for 5 weeks in chondrogenic differentiating conditions. We measured the effect of varying BSA concentrations on the chondrogenic potential of hASCs by monitoring pellet diameter weekly throughout the 5-week cultivation period. Notably, we observed an inverse relationship between pellet growth and BSA concentration, wherein lower BSA concentrations resulted in larger pellet sizes. However, the effect size varied among the different donors. Consequently, donors were categorized into two distinct responder groups based on their relative changes in size from d7 to d35. While all donors benefited from the lowest BSA concentration, three exhibited a positive relative size increase for all BSA concentrations, with the highest increase observed under LD (52%), followed by MD (28%), and HD (0.72%). These donors were designated as high responders. The remaining three donors shrank with all three BSA concentrations over the 5 weeks and were categorized as low responders. Hence, these donors resulted in negative relative growth, which was less pronounced for LD (−24.6%) compared to that for MD (−29.9%) and that for HD (−39.1%). However, one of the low responders showed increasing variability with decreasing BSA concentration with a broad range of pellet sizes in the LD group.

**Figure 1:**
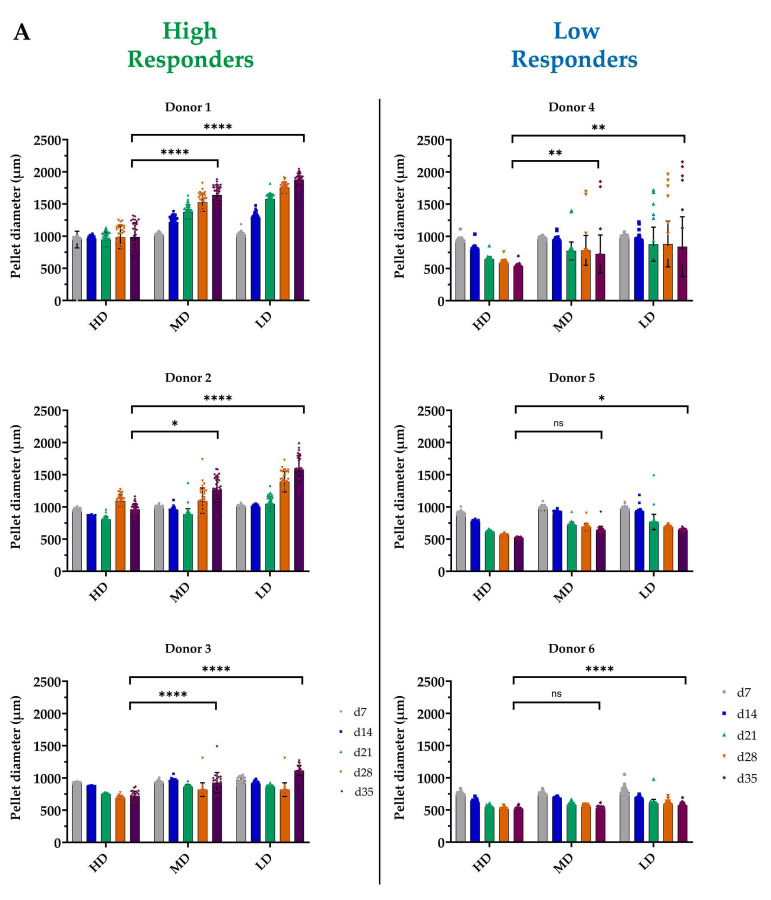

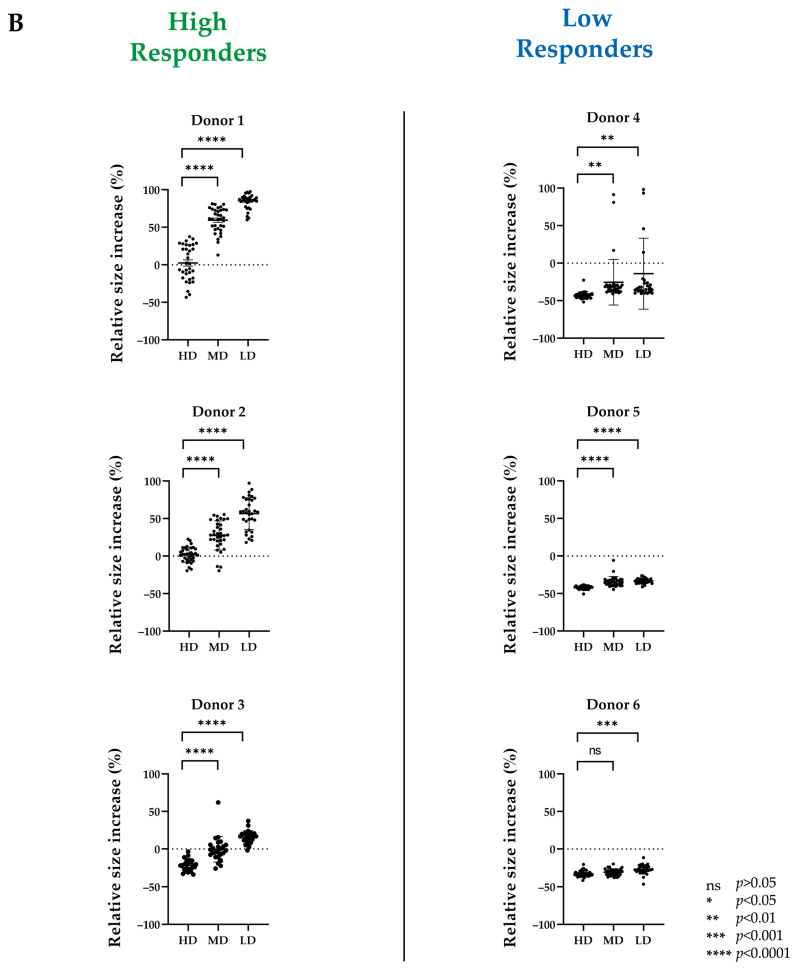
Effect of the three BSA concentrations (HD, MD, LD) on the resulting pellet size was assessed for each time point of the experiment (d7, d14, d21, d28, and d35; *n* ≥ 25). (**A**) Pellet size distribution over the cultivation time for each donor and (**B**) relative size increase for each independent BSA concentration. Donors were divided into two categories based on their response to the BSA dose: (1) high responders (donors 1, 2 and 3) were characterized by a general increase in pellet size and a dose-dependent response to BSA, most notably under LD, and a high intrinsic differentiation potential; (2) low responders (donors 4, 5, and 6) exhibited a minimal response to BSA, with pellets decreasing in size, and a lower intrinsic differentiation capacity. Statistical significance: **** *p* < 0.0001, *** *p* < 0.001, ** *p* < 0.01, * *p* < 0.05, ns *p* > 0.05.

In detail, the single donors responded as follows: (1) high responders: donors 1, 2, and 3 exhibited an increase in pellet diameter as BSA concentrations decreased. In donor 1, the most substantial increase occurred under LD (1024.15 ± 34.74 μm to 1879.51 ± 99.04 μm), followed by MD (1020.47 ± 25.50 μm to 1640.63 ± 153.13 μm). Both LD and MD increases were significantly higher than the increase observed under HD conditions (Figure 1A). HD media showed only a slight pellet size increase at later time points, resulting in a mere 2% increase, while MD media exhibited a 59.5% increase and LD media demonstrated an 85% increase, both significantly surpassing the HD media (*p* < 0.0001 HD vs. MD and *p* < 0.0001 HD vs. LD). Although donor 1 exhibited a consistent increase throughout the entire cultivation period, donors 2 and 3 experienced shrinkage at early time points, with donor 2 shrinking until the third week (except for under LD) and donor 3 until the fourth week. Nonetheless, under both LD and MD conditions, pellet sizes for donors 2 and 3 were significantly higher compared to the HD ones at the endpoint analyses (d35) (Donor 2: LD: from 1004.66 ± 24.27 μm to 1579.16 ± 214.80 μm; MD: from 948.22 ± 23.23 μm to 1266.77 ± 196.78 μm; *p* = 0.009 HD vs. MD; *p* < 0.0001 for HD vs. LD) (Donor 3: LD: from 964.47 ± 48.10 μm to 1114.89 ± 80.02 μm; MD: from 931.70 ± 27.45 μm to 928.04 ± 154.52 μm; *p* < 0.0001 for HD vs. MD; *p* < 0.0001 for HD vs. LD) (Figure 1A). Considering the earlier indicated shrinkage, the relative growth increase for donor 2 under LD was 56% and for donor 3 it was 15.7%, both significantly higher than that under HD (*p* < 0.0001 HD vs. MD; *p* < 0.0001 HD vs. LD) (Figure 1B).

(2) Low responders (donors 4, 5, and 6) exhibited a diminished performance characterized by a reduction in pellet size over the cultivation time and a low response to BSA concentration. Donor 4 was characterized by a very high variability with a reduced BSA concentration (LD and MD), with some pellets reaching nearly double the mean size. Nevertheless, as for the donors 5 and 6, it was characterized by a general reduction in the mean pellet size, which was significantly lower at d35 in the LD (976.63 ± 26.37 μm to 839.36.51 ± 464.96 μm) and MD (975.56 ± 18.93 μm to 725.80 ± 293.98 μm) groups than in the HD group (*p* = 0.0016 HD vs. MD; *p* = 0.0012 HD vs. LD) (Figure 1A). The pellets from this donor decreased by 13.9% under LD, 25% under MD, and 42% under HD (*p* = 0.0055 HD vs. MD; *p* = 0.0025 HD vs. LD) (Figure 1B). Despite the overall shrinkage also observed in donors 5 and 6, the size difference between LD and HD was statistically significant (Donor 5: 972.54 ± 28.03 μm to 644.12 ± 23.47 μm; *p* = 0.047) (Donor 6: 781.01 ± 58.09 μm to 573.17 ± 38.09 μm; *p* < 0.0001) (Figure 1A). This resulted in a notably smaller size difference between LD and HD; donor 5 pellets decreased by 33.1% under LD and 42.2% under HD, while donor 6 exhibited a reduction of 26.7% under LD and 32.3% with HD (Figure 1B).

To summarize, a high BSA concentration hindered pellet growth, which was particularly evident in donors with an inherent potential for large pellet sizes (“good differentiating donors”), being especially notable among the high responders (donors 1, 2, and 3). Consequently, the optimal response is observed in the best donors under low BSA concentrations.

To assess the potential influence of varying proliferation capacities on the donor differences, microscopic images during the 2D stage preceding the pellet formation were taken, and the population doubling time (PDT) was calculated (Figure S1). From the high responders, donors 1 and 2 exhibited an identical PDT of 38 h 52 min. Donor 3 showed a slightly slower proliferation rate with a PDT of 41 h 20 min, yet all three donors displayed the densest confluence and most uniform appearance in the 2D culture. However, donors 4 and 5, from the low responders, had the same PDT as that of the first two high responders (38 h 52 min). The donor proliferating slowest was donor 6 with a PDT of 46 h 30 min, characterized by a distinctive elongated morphology with extended filopodia, contrasting with the typical spindle-like morphology observed in the other donors.

### 3.2. BSA Modulates Matrix Formation in a Concentration- and Donor-Dependent Manner

Similar to the pellet size analysis, in histology, the size of the pellets was very different, hence, they were stained with Azan to visualize the variability in cell density. In general, the tendency reflected that the smaller undifferentiated pellets had a higher number of cells (Figure S2). Regarding the morphology, cells appeared rounded in the center and more elongated on the surfaces.

**Figure 2:**
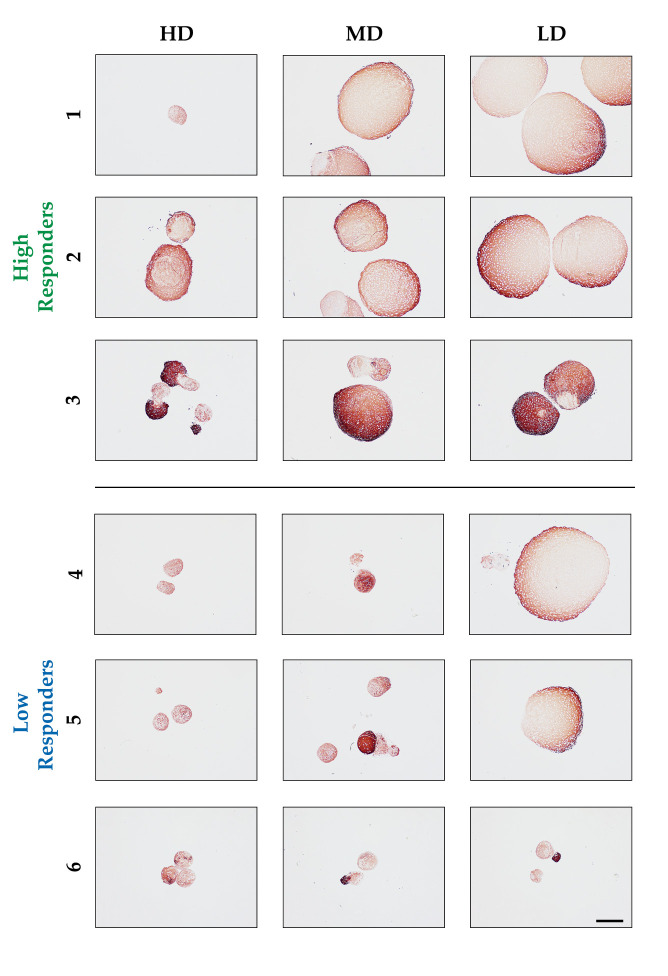
(Immuno-)histochemical stained sections of the different pellets from each donor under each BSA concentration after 5 weeks in culture, stained for collagen type II. High responders (donors 1, 2, and 3) exhibited larger pellet diameters and a more homogeneous positive staining indicative of a hyaline-like matrix. However, the displayed pellet from donor 1 is unrepresentative small (as known from the pellet size data), but the rest of the pellets were lost during histological processing. The low responders (donors 4, 5, and 6) generally displayed a smaller size (HD and MD), and only for donors 4 and 5 was a hyaline-like collagen type II matrix under LD visualized. Adjacent small pellets and some specific areas within MD and LD for donor 6 exhibited intense staining for collagen type II. (μ-bar: 500 μm).

**Figure 3:**
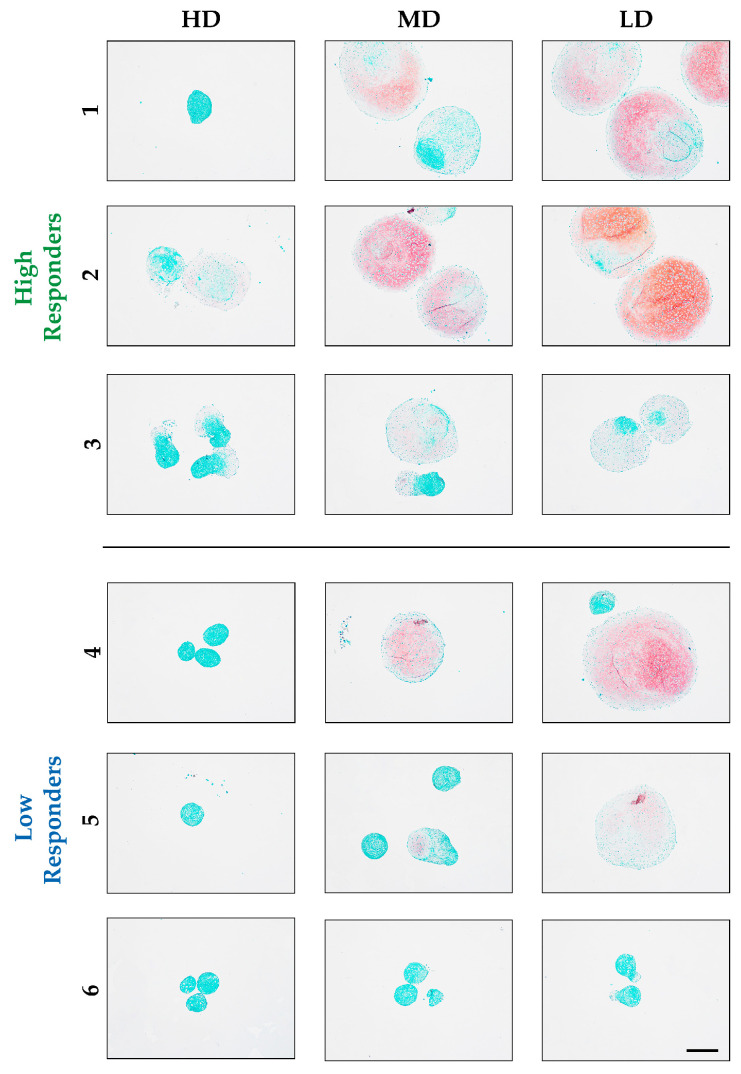
Comparison of the two donor groups according to the BSA concentration for GAG content. Saf O staining of histological sections showed a cartilage-like matrix with intense GAG staining for donors 1 and 2 from the high responders for MD and LD BSA. Only those pellets with a significantly bigger size were positively stained in the high responders (donors 1 and 2) as well as in donors 4 and 5 from the low responders, especially at LD. However, the small pellets from the low responders (donors 5 and 6), along with the high responders exhibiting minimal pellet growth (donor 3), showed limited presence of GAGs. (μ-bar: 500 μm).

To qualitatively evaluate the chondrogenic differentiation of the hASCs pellets according to each BSA concentration, (immuno-)histological analysis was performed. The observed increase in pellet size for the high responders correlated with a hyaline matrix that was positive for collagen type II (MD and LD) (Figure 2). Among the high responders, donors 1 and 2 demonstrated a more fibrocartilage-like appearance when induced under the highest BSA concentration. Donor 3 had medium pellet sizes, generally smaller than those of the previous donors, especially in the HD and MD BSA conditions. Nevertheless, all pellets from this donor showcased an exceptionally intense collagen type II staining, likely attributed to the denser packing of collagen type 2 and more accessibility of the antibody due to low concentrations of GAGs (see Figure 3). Additionally, within or next to the positively stained areas of the pellet, there were collagen type II-negative areas, but the ratio of stained to unstained areas was higher at lower BSA concentrations. The low responders (donors 4, 5, and 6) had pellets that were smaller overall compared to those of the other donors and appeared fibrous, especially in the MD and HD conditions. For donors 4 and 5, the large LD pellets were collagen type 2-positive with a hyaline matrix, similar to those of the high responders. Notably, the adjacent small pellets (donor 4), measuring approximately 18% of the large ones, of the same group (i.e., the same section) were almost collagen type 2-negative. In the MD pellets from donors 5 and 6, as well as in the LD ones of donor 6, remarkably intense collagen type II staining (similar to that of donor 3) within a generally negative matrix was observed. These areas were larger in donor 5 than in donor 6. However, individual pellets were larger and homogenously stained with more intense and darker brown (as in donors 1 and 2).

In general, the high responder group exhibited an increased collagen type II deposition with lower concentrations of BSA, whereas the low responder group appeared to differentiate towards a more fibrotic phenotype with only focal areas of differentiation in otherwise undifferentiated pellets, but there was a hyaline-like matrix under LD conditions for two donors (see Figure 2).

Pellets were also stained for collagen type 1 to evaluate non-chondrogenic differentiation. In all cases, they were positive and uniformly stained for collagen type 1, as visualized by immunohistochemistry (Figure S3). The high responders were also positively stained but were less intense, especially for the LD BSA from donor 1 and the MD BSA from donor 2. The most intense staining was observed in the smallest pellets of the low responders, particularly with the HD and MD BSA concentrations, indicating an elevated presence of collagen type 1.

Glycosaminoglycans (GAGs) are the second most important extracellular matrix (ECM) constituent of hyaline cartilage and are responsible for the retention of water to support the compressive forces on the tissue. Therefore, GAG analysis is of extreme importance. Pellets were stained for Safranin O (Saf O), a cationic dye used to detect proteoglycans, which stains newly synthesized GAGs in red (Figure 3). Following collagen type II, donors 1 and 2 from the high responders, which had a larger diameter and more homogeneous collagen type 2 staining, also exhibited an increased GAG deposition for MD, especially for LD BSA. However, donor 3, despite its growth and collagen type 2 positivity, was negatively stained for Saf O. The intense collagen type 2 staining (Figure 2) might be related to the absence of GAGs, leading to a denser packing and less masking of collagen type 2. Low responders (donors 4, 5, and 6) were overall negatively stained with Saf O, except for some areas in the MD and LD condition of donors 4 and 5, which also stained for collagen type II, and donor 4 showed a more intense staining in the large pellets. The absence of staining is supposedly a result of their poor differentiation capacity and lack of response to BSA.

Similar results were observed using Alcian blue, where LD and MD from donor 1 and donor 2 of the high responders and some pellets of donor 4 from the low responders under LD had the highest positive staining, characterized by bright blue color. The small pellets and, in general, the low responders, particularly donors 5 and 6, were negatively stained (Figure S4).

### 3.3. Reduced Concentration of BSA Supports Osteogenic Differentiation

**Figure 4:**
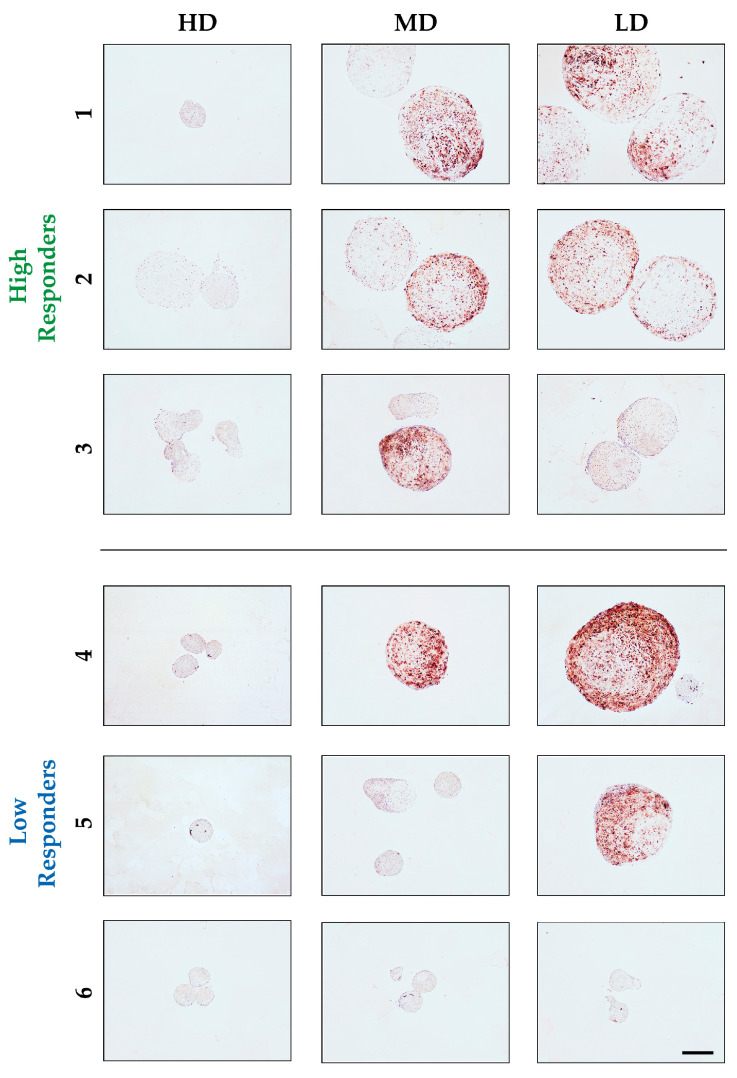
Effect of BSA concentrations on the hypertrophic and osteogenic phenotype of the different donors, as visualized on osteocalcin-stained sections. Overall, all pellets under the HD condition were mainly negatively stained. However, the larger pellets from the high responder group (donors 1–3) were positively stained for osteocalcin under MD and LD BSA. Similar behavior was observed for the large pellets of donor 4 under MD and LD, as well as for donor 5 under LD, from the low responders. The rest of the pellets from the low responder group (i.e., donor 6) were negatively stained for OCN. (μ-bar: 500 μm).

To assess the potential impact of BSA on the osteogenic and hypertrophic phenotype of hASCs during chondrogenic differentiation, immunohistochemical analysis of osteocalcin (OCN) was conducted (Figure 4). Interestingly, osteocalcin exhibited a positive correlation with the presence of collagen type 2, as indicated by increased OCN deposition with decreasing concentrations of BSA, with all donors being negatively stained for osteocalcin with HD BSA. However, the intra- and inter-pellet variability for OCN was higher than that for collagen type II. Both donors 1 and 2, belonging to the high responder group, exhibited positive staining for pellets under both MD and LD BSA. Surprisingly, under the LD BSA condition, donor 3, also classified as a high responder, showed no osteocalcin deposition, whereas the MD group demonstrated varying levels of intensity. Similarly to the first two donors, donor 4 from the low responders followed the same behavior with increased staining exclusively in the large pellets of MD and LD BSA, with even higher intensity than that of the high responders. As illustrated in Figure 4, the small pellet adjacent to the larger one in the LD condition showed negative staining for OCN. Likewise, in the small pellets of donors 5 and 6, no OCN was deposited, and only the exceptionally large pellet from donor 5 under LD was stained positively for OCN in the outer areas.

To further elucidate the progression of terminal differentiation into a hypertrophic phenotype, pellets were stained for collagen type X (Figure S5), matrix metalloproteinase-13 MMP-13 (Figure S6), and MMP-3 (Figure S7). While collagen type X was only faintly stained in the differentiated pellets, MMP-3 and MMP-13 were rather localized in the undifferentiated ones.

### 3.4. Glycosaminoglycan Content Correlates with Histology and Is BSA- and Donor-Dependent

**Figure 5:**
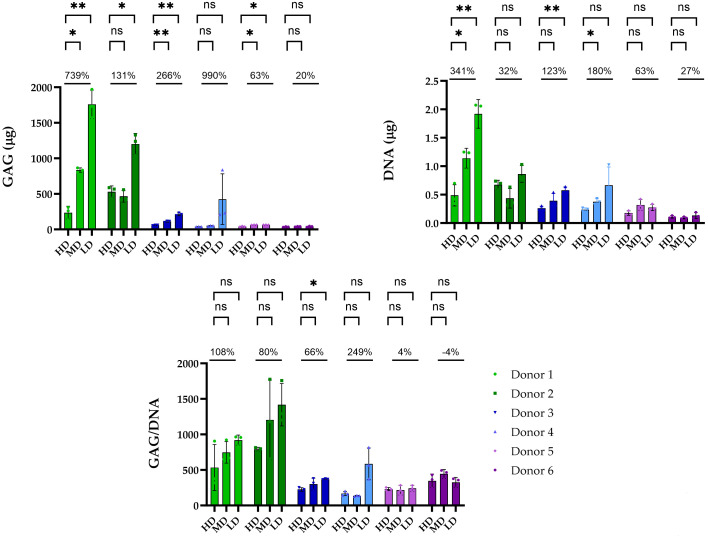
Quantitative GAG and DNA content analysis after the cultivation period (d35) for each donor under the different BSA concentrations. Similarly to histological findings, a positive trend could be observed for high responder donors, which had higher GAG and DNA contents under the LD BSA concentration, with substantially lower GAG content being observed for donor 3. From the low responders, only donor 4 followed a similar pattern with a higher GAG, DNA, and GAG/DNA ratio under the LD condition, but, overall, they demonstrated limited GAG synthesis and were unresponsive to varying BSA concentrations. * *p* < 0.05; ** *p* < 0.01; ns *p* > 0.05.

At the endpoint of the experiment, the GAG content was quantified (d35) to account for the total glycosaminoglycan and DNA content via the DMMB and Picogreen assays. Quantitative analysis of GAGs was consistent with the histological images, revealing clear differences among the BSA concentrations for the different responder groups (Figure 5). For the absolute GAG concentration, again, donors 1 and 2 from the high responders, showed the highest value. For all three high responders, the LD BSA condition produced significantly higher total GAG content than the HD one. (Donor 1: LD: 1758 ± 201.5; MD: 834.7 ± 30.55; HD: 236.0 ± 52.49 μg; *p* = 0.0022 LD vs. HD; *p* = 0.0138 HD vs. MD. Donor 2: 1203 ± 147.4 μg, MD: 465.51 ± 81.25 μg; HD: 531.1 ± 81.93 μg; *p* = ns LD vs. MD; *p* = 0.0110 LD vs. HD. Donor 3: HD: 58.49 ± 3.94 μg; MD: 110.52 ± 9.97 μg; LD: 212.65 ± 19.51 μg; *p* = 0.0062 HD vs. MD; *p* = 0.0090 HD vs. LD). As demonstrated earlier with Safranin O staining, only a low amount of GAGs was detectable in donor 3. From the low responders, similarly to the histological results, donor 4 showed the highest GAG content under the LD condition but with very high variability as previously shown. However, non-significant differences were found among them. Despite no obvious Saf O or Alcian blue staining, donor 5 from the low responder group showed a significant increase in GAGs for LD and MD BSA compared to HD BSA (donor 4: LD: 212.7 ± 19.51; MD: 110.5 ± 9.97; HD: 58.49 ± 3.94; *p* = 0.0062 HD vs. MD; *p* = 0.0090 HD vs. LD) (donor 5: LD: 64.54 ± 0.73; MD: 64.34 ± 2.28; HD: 40.09 ± 5.97; *p* = 0.0126 HD vs. MD; *p* = 0.0319 HD vs. LD). For donor 6, GAG quantifications were in line with histology since no significant variations in the GAG content were observed across different BSA concentrations.

Nonetheless, the absolute GAG content is dependent on the pellet size and, consequently, on the total DNA content. Notably, the DNA content was significantly higher for donor 1 (LD BSA vs. HD BSA *p* = 0.0025; MD BSA vs. HD BSA; *p* = 0.0210), for donor 3 within the high responder group (LD vs. HD BSA; *p* = 0.0075), and for donor 4 from the low responders in the MD condition (MD BSA vs. HD BSA; *p* = 0.0075) (Figure 5). When quantifying the cellular-level synthesis of GAGs, the influence of BSA was less pronounced when compared to the total GAG content. Despite the GAG/DNA ratio notably being the highest within the high responder group, non-significant differences were found among the BSA concentrations for donors 1 and 2, probably due to the high variability. Donor 3 displayed a significantly higher GAG/DNA ratio with LD in comparison to HD (*p* = 0.0184), despite a considerably lower GAG/DNA ratio compared to that of the other two donors. Non-significant differences were found for the low responders (Figure 5). However, similarly to the absolute GAG content, the ratio was considerably higher under LD conditions, especially for donor 4, a trend that was also evident in the Saf O staining (Figure 3).

In summary, consistent with histological findings, the lowest BSA concentrations exerted a more pronounced effect on donors with a superior intrinsic differentiation potential (high responders). These donors exhibited elevated GAG content under LD conditions.

### 3.5. Gene Expression of Matrix Marker Does Not Show the BSA-Dependency Found at the Protein Level

**Figure 6:**
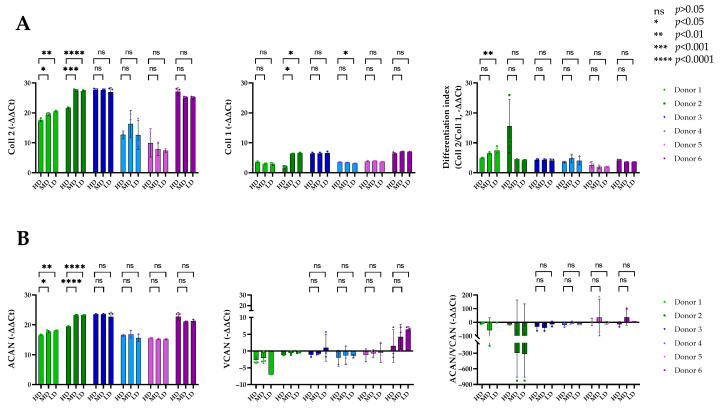
Quantitative endpoint analysis of collagen type II, I, aggrecan, and versican gene expression (RT-PCR). (**A**)*. Coll 2* was significantly higher for the high responders with MD and LD BSA. *Coll 1* expression was very similar for all the donors, with higher expression for donors 2, 4 and 6. (**B**). Similarly to *Coll 2*, *ACAN* was significantly higher for donors 1 and 2 from the high responder group both at MD and LD. *VCAN* expression was, conversely, negative in all cases except for that of donor 6, suggesting a more dedifferentiated phenotype for the low responders. * *p* < 0.05; ** *p* < 0.01; *** *p* < 0.001; **** *p* < 0.0001.

To compare the differentiation capacity of the pellets under each BSA concentration, they were analyzed at the endpoint (d35) by RT-PCR for the most common chondrogenic markers, collagen type II (*Coll 2*), and aggrecan (*ACAN*), and its expression was compared to collagen type I (*Coll 1*) and versican (*VCAN*), which are markers of dedifferentiation (Figure 6). *Coll 2* and *ACAN* expression followed a very similar expression pattern for all donors and were significantly higher for donors 1 and 2 within the high responder group for both LD BSA and MD BSA compared to HD BSA. (Donor 1 *Coll2*: *p* = 0.0450 HD vs. MD; *p* = 0.0063 HD vs. LD. Donor 2 *Coll2*: *p* = 0.0003 HD vs. MD; *p* < 0.0001 HD vs. LD. Donor 1 *ACAN*: *p* = 0.0370 HD vs. LD; *p* = 0.0014 HD vs. LD. Donor 2 *ACAN*: *p* < 0.0001 HD vs. MD and HD vs. LD.) We could not observe any significant differences between the BSA concentrations at the expression level of *Coll 2* and *ACAN* for donor 3. In the case of the low responders, non-significant variations were observed among BSA concentrations for *Coll 2* and *ACAN*, although their expression levels were higher under the MD for donor 4, as well as under the HD condition for donors 5 and 6. In summary, the high responders (donors 1, 2 and 3) had a generally high expression level of Coll 2; donors 4 and 5 from the low responder group showed a rather low expression, while donor 6 had a very high expression, which was even higher than that of donor 1 (Figure 6A).

*Coll 1* expression was consistently lower across all the donors compared to that of *Coll 2.* Among the high responders, donors 1 and 3 showed non-significant differences between the BSA concentrations, with slightly lower values observed for LD in donor 1. However, *Coll 1* was significantly higher in expression for donor 2 under MD and LD concentrations compared to that under HD. Overall, expression levels were generally similar across all groups, with donor 1 closely resembling donors 4 and 5. Additionally, donor 3 exhibited similarities to donor 6, being slightly higher under MD. The ratio of *Coll 2*, the marker for differentiated chondrocytes in hyaline cartilage, and Coll 1, the marker of dedifferentiated chondrocytes, fibroblasts and osteoblasts served as the cell differentiation index. This reflected a similar trend to the one previously described. Donor 1 from the high responders was the best differentiator, aligning with the histological and pellet size findings, particularly under the LD concentration. In the other two high responders, non-significant differences were found among the concentrations, with a considerably higher differentiation index under HD for donor 2. The low responders showed non-significant differences in the differentiation index among the different BSA concentrations. Similarly, for *VCAN*, for these donors, non-significant differences were observed among the different BSA concentrations, despite donor 6 displaying the highest concentration of *VCAN* (Figure 6B). Statistical tests could not be computed for donors 1 and 2 due to insufficient detection. Correspondingly, non-significant differences were found in the *ACAN/VCAN* ratio.

In summary, only donor 1 showed an increase in chondrogenic marker gene expression with decreasing concentrations of BSA (differentiation index). Few differences were found among the low-responder donors with a more osteogenic and hypertrophic phenotype.

## 4. Discussion

Albumin is one of the most important proteins in the human body with a vast range of functions that have an impact on the cellular, physiological, and tissue levels [36]. In the biomedical field, it is used as a local therapeutic agent, biomaterial (e.g., drug-delivery system), carrier, and as a media supplement in vitro [36,37]. This study explored the role of albumin as a supplement in a chondrogenic differentiation medium for hASCs, aiming to facilitate the transition to a xeno-free medium and avoiding substances of natural origin with high charge variability while preserving the chondrogenic phenotype. Surprisingly, we found that a diminished concentration of BSA (from 1.25 to 0.125 and 0.0125 mg/mL) resulted in enhanced pellet formation. Notably, the reduced concentrations are 10 and 100× lower than our standard concentration, which is the typical concentration utilized for chondrogenic differentiation, as described by several research groups, ranging from around 1–1.25 mg/mL [38,39,40]. For this study, we utilized hASCs obtained from liposuctions of six distinct donors and classified them based on their responses to the three BSA concentrations. The high responders exhibited superior chondrogenic differentiation (i.e., histologically and with regard to GAG content) with a reduced BSA concentration. The low responders were characterized by general shrinkage and smaller pellet sizes but still benefited from a reduced BSA concentration.

In donors 5 and 6 from the low responders, as well as donor 3 from the high responders, areas with differential collagen type II staining were identified as subpopulations generating small outgrowth areas situated either adjacent to or at the periphery of the main pellets. Previous studies have identified distinct cell populations or subsets within ASC cultures, characterized by varying proliferation and differentiation capacities, potentially contributing to the observed heterogeneous differentiation within certain areas of the pellets [41,42,43]. To further validate the differences in the proliferation of subpopulations, additional fluorescence-activated cell sorting (FACS) could be performed. This would enable the analysis of different surface markers for each cell population within a single pellet [44,45].

Although pellet size, histology, and GAG content data were consistent, we noted discrepancies at the gene expression level, which was considerably lower than that which was expected from both histology and GAGs, particularly in the high responders and in donor 6 from the low responders with a remarkably high *Coll 2* expression. Since PCR data are relative data per cell, while pellet diameter and histology are absolute data per pellet, the lower effect on gene expression might indicate that protein synthesis is accomplished by a higher number of cells rather than a higher synthetic activity of the individual cells. Consequently, BSA may either inhibit chondrogenic progression or its absence might stimulate the proliferation of chondrogenic sublines. Support for a higher cell number in MD and LD pellets comes from DNA quantification and the higher GAG content when comparing total pellets to GAG/DNA.

Generally, all donors benefitted from lowering the BSA concentration, a phenomenon not previously reported. This observation contrasts with the existing literature that suggests the opposite effect. For instance, Lund et al. reported that hASCs could not differentiate into the chondrogenic lineage using two distinct serum-replacement media, both containing human and bovine serum albumin. Similarly, Baptista et al. observed the incomplete differentiation of hASCs, with only specific areas stained for Saf O, using a dose of 1.25 μg/mL—10× smaller than the LD in this study [46]. Considering its primary function as a carrier or binding protein to molecules such as hormones, drugs, or growth factors [36,47], the observed effects could be attributed to several factors, and an adverse effect could arise from following points: (1) BSA may actively bind the growth factors in the media (e.g., TGF-β3, BMP-6), preventing them from acquiring their active isoform and binding to their receptor. Some authors have considered albumin as a molecular chaperone, preventing misfolding and/or aggregation of other proteins [48]. However, studies like Wan et al. have used HSA as a stabilization agent combined with the TGF-β1 receptor by a fusion protein to improve therapeutic potential [49]. (2) As a natural product, BSA may carry ligands with anti-chondrogenic effects on hASCs. For instance, BSA has been used as a transport protein of molecules such as Interleukin-2 (IL-2), vascular endothelial growth factor (VEGF), or interferon-γ (IFN-γ) [50,51]. (3) BSA may adhere to cell surfaces, potentially impeding the receptor-binding of relevant molecules such as growth factors.

However, among the positive effects reported, BSA antioxidant properties are known to prevent cell damage caused by the oxidative processes and Reactive Oxygen Species (ROS) produced in a cell culture vessel or bioreactor. The generation of ROS is created by the interaction of the medium components, high oxygen tension, and general cell metabolism [20,52,53]. Albumin can bind metal ions such as copper (Cu^2+^), which catalyzes the production of free radicals, or iron (Fe^2+^) to prevent these damaging ROS or scavenge free radicals released in the medium [7,54,55,56,57,58,59]. Despite these antioxidant properties, BSA is also known to exist in different redox states, and its oxidized state could adversely affect chondrogenicity.

The observed response to BSA and subsequent chondrogenic differentiation in our donors was highly dependent on their differentiation potential. The different responder groups displayed different intrinsic differentiation potentials and, interestingly, the good donors benefitted the most (donors 1, 2, and 3). When it comes to donor variability, additional factors, including donor characteristics such as age and sex, are known to significantly influence hASC proliferation and differentiation. Several studies have demonstrated that elderly donors (>60 years) exhibit slower proliferation and impaired chondrogenic differentiation, while female hASCs displayed increased variability and less multipotency potential, particularly in younger donors [60,61,62]. Moreover, the anatomical region for harvesting, liposuction method, and isolation technique were reported to affect the quality, functionality, plasticity, and differentiation potential of hASCs [63]. Investigations by Fraser et al. and Qu et al. highlighted differences in the yield and number of nucleated cells on adipose tissue harvested from the hip and thigh [64,65]. The multilineage potential has also been associated with the liposuction aspiration method, despite some authors like Duscher et al. having demonstrated an equivalent yield, viability, and similar chondrogenic capacity between ultrasound-assisted liposuction and standard suction-assisted lipoaspiration [66]. In this study, female donors between the ages of 30 and 55 were included, and the cells were obtained through power-assisted liposuction, a more precise method than that of the standard routine, maintaining the methodical preconditions as standardized as possible.

As part of their multilineage potential, hASCs can also undergo osteogenic differentiation, acquiring a hypertrophic and osteogenic phenotype. Therefore, we also evaluated bone-related and hypertrophic proteins such as osteocalcin as well as collagen type I and X [67]. Donors followed a similar trend as that for collagen type II, showing increased variability between pellets for OCN than for collagen type II, especially among the high responders. Osteocalcin is a calcium-binding extracellular matrix protein, expressed both by osteoblasts and hypertrophic chondrocytes during the process of endochondral ossification [68,69,70,71]. In this study, the increased synthesis of osteocalcin and collagen type I with lower BSA concentrations corresponds to hypertrophy, a process which inversely seems to be prevented by BSA. However, for the late hypertrophic markers such as collagen type X, only very faint staining was visualized, suggesting the onset of a more advanced hypertrophic stage. MMP-13 and MMP-3 were positive only in some undifferentiated pellets or areas, indicating a higher turnover of collagen in those pellets The growth factors supplemented in the media in vitro (i.e., TGF-β3 and BMP-6) are crucial for inducing a chondrogenic phenotype but can ultimately lead to terminal differentiation, upregulating hypertrophic markers such as collagen type X, alkaline phosphatase (ALP), Runt-related transcription factor 2 (RUNX2), as well as matrix metalloproteinases (e.g., MMP13) and VEGF [72,73,74]. Despite its potential drawbacks, hypertrophy has been used in tissue engineering and regenerative medicine to grow bone since it can produce unstable hypertrophic chondrocytes that progress to the bone in vivo through endochondral ossification [74,75].

However, for cartilage regeneration, hypertrophy is an issue, especially when using MSCs. Therefore, several approaches have been investigated to circumvent the hypertrophy of hASCs. The inclusion of chondrogenic factors such as mechanical stimulation, hypoxic cultures, co-cultures with chondrocytes, encapsulation into hydrogels, and even adenoviral transfection with chondrogenic-inducible factors such as SOX9 have been shown to promote chondrogenesis with reduced hypertrophy [75,76,77,78]. Additionally, the cell number per pellet was found to be critical for the optimal chondrogenic differentiation of MSCs. Several authors have reported an increase in hypertrophic and osteogenic markers when culturing 200,000 cells/pellet under chondrogenic conditions, whereas micro-pellets of 5000 cells/pellet resulted in more hyaline-like cartilage pellets [79]. Since we used large pellets with 200,000 cells/pellet in this study, further investigations with a reduced number of cells/pellets would be interesting to elucidate if a reduction in BSA has a similar effect on hypertrophy in micro-pellets [38,70,80].

To the best of our knowledge, an anti-chondrogenic effect of BSA, as found in this study, has not been reported yet. In contrast, albumin or albumin-containing body fluids have been used for therapeutic reasons such as the treatment of osteoarthritis (OA). Bar-Or D. et al. showed that the addition of a low molecular weight fraction of commercial 5% HSA could induce chondrogenic differentiation in BM-MSCs in spheroids [26]. This compound is currently undergoing clinical trials for OA and pain management due to its anti-inflammatory properties. Platelet-rich plasma (PRP) containing albumin is also used in clinical routines to alleviate OA. However, the concentration of albumin and the different proteins in the PRP, as well as the mechanism of action of both BSA and its ligands, remain incompletely understood. This lack of understanding could potentially contribute to variations observed in isolated PRP. Nonetheless, it has been shown that PRP promotes articular cartilage regeneration and the chondrogenic differentiation of ASCs in vitro [81,82]. Furthermore, albumin, being the most abundant protein in the synovial fluid, plays a key role in joint lubrication and the transportation of small molecules in different tissues of the joint [83,84]. Changes in albumin concentrations in the synovial fluid are often associated with inflammatory conditions such as OA or rheumatoid arthritis, with levels typically decreasing, rendering it a potential biomarker in clinical settings [85,86]. The incomplete and controversial information regarding the effect of albumin on chondrogenesis and joint homeostasis needs further investigation into its natural role and variations in healthy and pathologic joints as well as how it may be a potential therapeutic agent.

The primary limitation of our study is the absence of a group completely devoid of BSA to assess if the trend of improved chondrogenicity and hypertrophy continues or if there is a lower limit or even a reversion of the effect. While we did not perform a mechanistic study to elucidate the observed effects due to its complexity, the narrative state of this information holds significance. It serves as valuable background knowledge for the design of in vitro and in vivo experiments, which is particularly relevant for studies dealing with synovial fluid and MSCs, offering essential context for researchers to consider.

## 5. Conclusions

In summary, BSA concentration significantly influenced the chondrogenic potential of human ASCs in a pellet culture system. This effect was donor-dependent and evident in terms of pellet growth and matrix composition. Overall, donors with higher intrinsic chondrogenic potential benefitted most in terms of pellet growth, collagen type II, and glycosaminoglycan synthesis with the lowest BSA concentration, which is 100 times lower than the commonly used one in the literature. However, it is noteworthy that low BSA concentrations also allowed for hypertrophy in the pellets. Understanding the mechanism behind this dual effect holds the key to improving in vitro chondrogenicity for MSCs, allowing for more reproducible results that closely resemble the clinical scenario.

## Data Availability

The data presented in this study are available on request from the corresponding author.

## Supplementary Materials

The following supporting information can be downloaded at https://www.mdpi.com/article/10.3390/biom14040413/s1; Figure S1: Phase contrast microscopic images of the hASCs from the six donors, in P3 cultured in 2D, and the population doubling time before pellet formation; Figure S2: Overview AZAN staining to visualize cell morphology and distribution on the pellets; Figure S3: Immunohistochemical analysis of collagen type I in the pellets derived from the different donors and exposed to varying BSA concentrations; Figure S4: Glycosaminoglycans distribution in hASCs pellets under the different BSA concentrations; Figure S5. Histological staining of the late hypertrophic differentiation marker collagen type X; Figure S6: Late hypertrophic differentiation characterized by MMP-13 histological staining; Figure S7: Matrix Metalloproteinase-3 (MMP-3) histological staining under the different BSA concentrations to further characterize the hypertrophic and osteogenic differentiation of the pellets.
