# Supplementary material for: Unlocking Potential: Low Bovine Serum Albumin Enhances the Chondrogenicity of Human Adipose-Derived Stromal Cells in Pellet Cultures
Source: Biomolecules. 2024 Mar 28;14(4):413. doi: 10.3390/biom14040413 (PMC11048491; doi:10.3390/biom14040413)
Supplement: Supplementary file 1 [file biomolecules-14-00413-s001.zip › biomolecules-2905429-supplementary.pdf]

biomolecules

Article

Unlocking Potential: Low Bovine Serum Albumin Enhances the Chondrogenicity of Human Adipose-derived Stromal Cells in Pellet Cultures

Isabel Casado-Losada † [1,2,4], Melanie Acosta † [1,2,4], Barbara Schädl [2,3], Eleni Priglinger [2,4,5], Susanne Wolbank [2,4], and Sylvia Nürnberger [1,2,4*]

Supplementary Materials

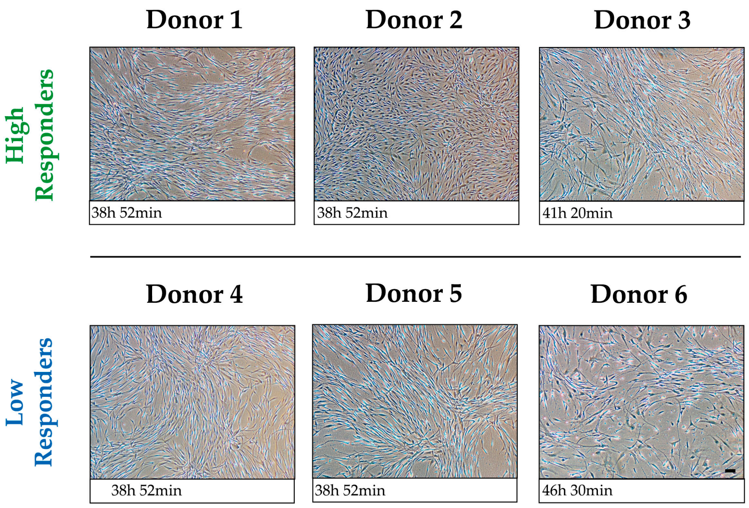

Figure S1. Phase contrast microscopic images of the hASCs from the six donors, in P3 cultured in 2D, and the population doubling time before pellet formation. All donor cells had a similar morphology characterized by elongated spindle-like cells, branching together and uniformly aligning with each other. Donor 6 from the low responders, showed the slowest proliferation, indicated by a PDT of 46h 40 mins, and a less dense appearance (μ-bar: 100 μm).

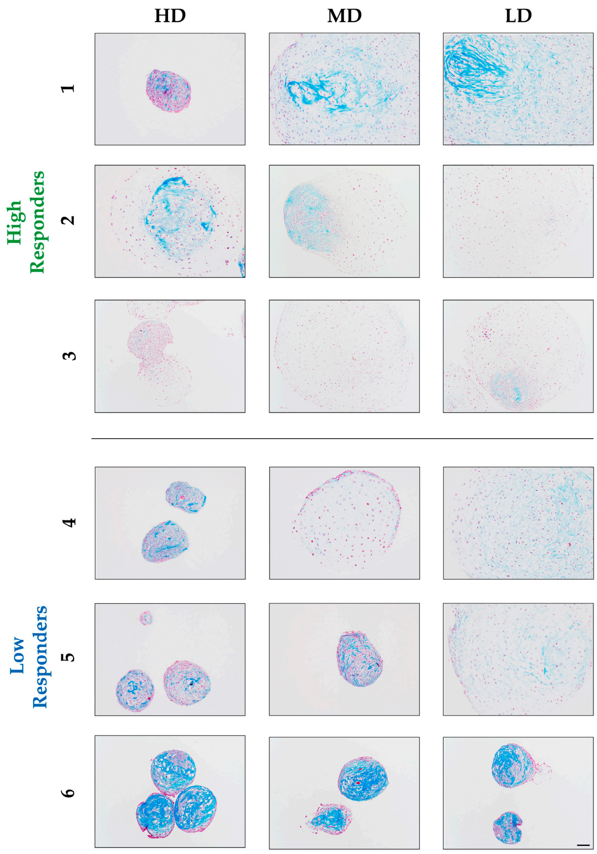

Figure S2. Overview AZAN staining to visualize cell morphology and distribution within the pellets. In the undifferentiated (collagen II negative) areas or pellets, more red staining, indicative of cell nuclei was observed. These pellets contained more elongated cells, whereas the differentiated hASCs appeared more rounded, resembling chondrocytes. Besides, the superficial cells were aligned along the pellet surface and were more frequently found as well in the un- or low-differentiated pellets. (μ-bar: 100 μm).

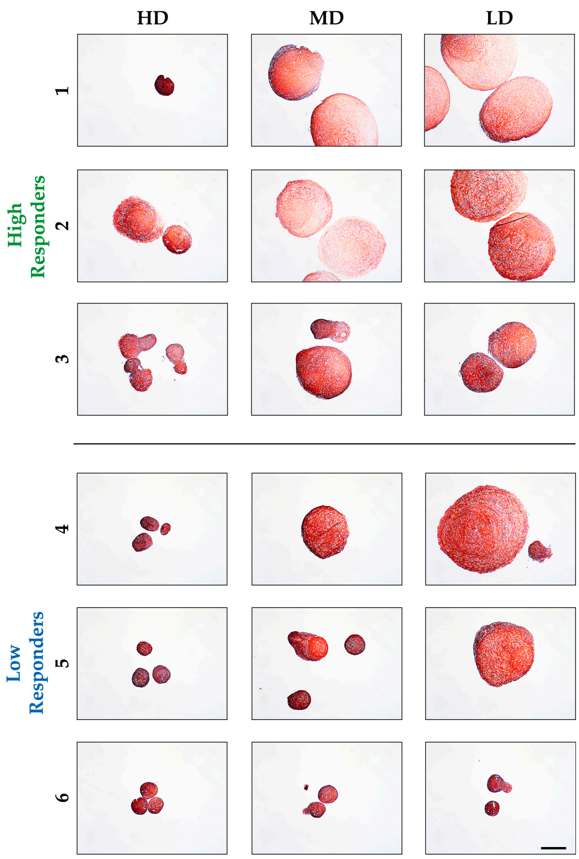

Figure S3. Immunohistochemical analysis of collagen type I in the pellets derived from the different donors and exposed to varying BSA concentrations. While all donors were positively stained for collagen type I, remarkably darker staining was evident under HD BSA, particularly in the smaller pellets of the low responders. In contrast, donors 1 and 2 from the high responders displayed less intense staining under MD and LD BSA conditions compared to the uniform staining observed in other donors and conditions (μ-bar: 500 μm).

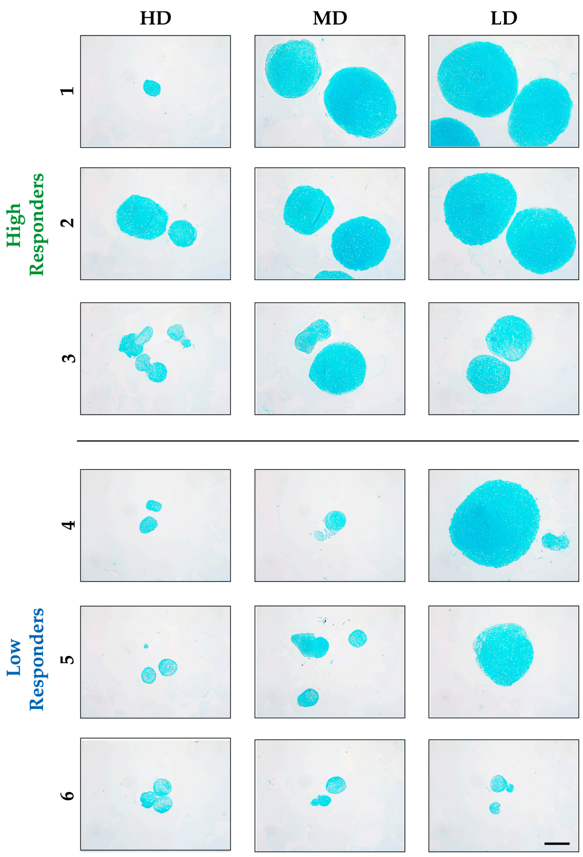

Figure S4. Glycosaminoglycans distribution in hASCs pellets under the different BSA concentrations. Alcian Blue staining was performed to visualize GAGs in all chondrogenic pellets, where bright blue indicates positive areas with lower GAGs and darker blue increased amounts. Positive staining was uniformly observed in large pellets, with donors 1 and 2 (high responders) exhibiting more intense staining under LD. For donor 3 (high responder), the staining was less uniform, with areas showing reduced GAGs under both LD and MD, the latter presenting darker blue. Reduced GAG content was evident under HD for all donors, especially for the low responders, characterized by less intense staining. Nevertheless, donor 4 displayed a significantly intensified bright staining under LD in the large pellet, while the small pellet was less intensely stained with a darker blue indicating a low number of GAGs. Additionally, in the low responders differential stained areas were found, particularly in the edges and adjacent outgrowth pellets, with a more pronounced staining (μ-bar: 500 μm).

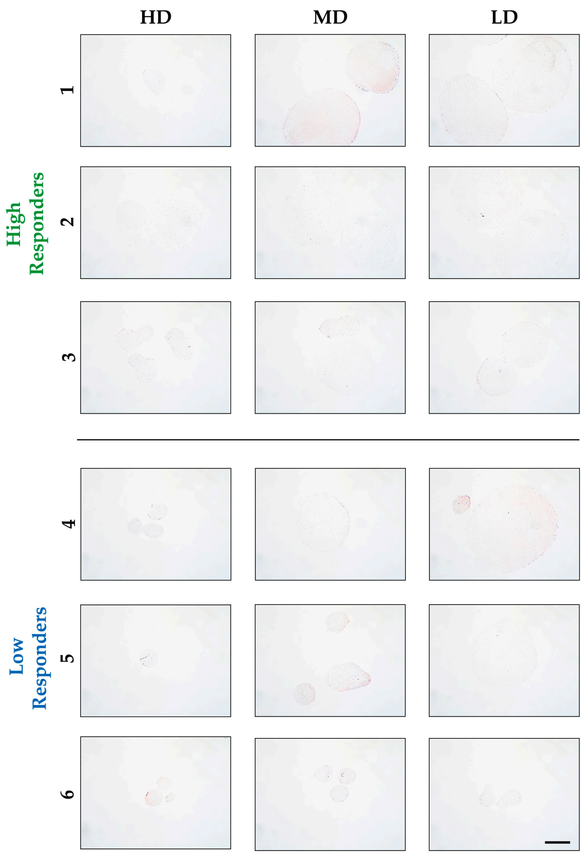

Figure S5. Histological staining of the late hypertrophic differentiation marker, Collagen type X. In general, all pellets were very faintly stained. However, there were slight increases in the staining intensity for donor 1 under MD BSA and donor 3 in HD from the high responders. Conversely, donor 2 was negatively stained across all concentrations. Among the low responders, donors 4 and 5 exhibited slight staining under LD and MD, respectively, with increased intensity observed on the small pellets. Donor 6 displayed a minor increase in the staining intensity under HD (μ-bar: 500 μm).

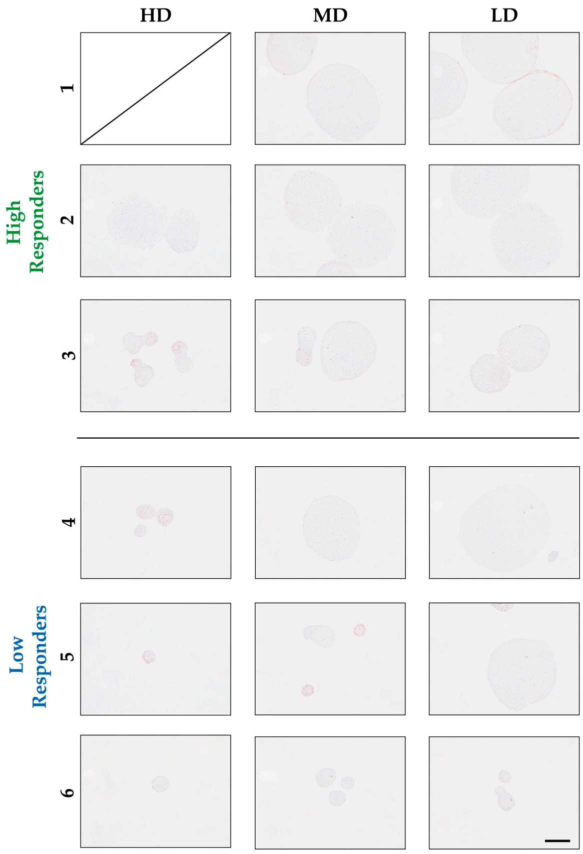

Figure S6. Late hypertrophic differentiation characterized by MMP-13 histological staining. Generally, light staining was visualized for all donors under the three BSA concentrations. However, some slight differences were observed on the high responders, especially for donor 1 under LD and donor 3 under HD, restricted to the surface of the pellets, with increased staining also for MD and LD on the central areas of the pellets. Donor 2 was negative for all concentrations. From the low responders, a more intense staining was observed for donor 4 under HD, and particularly for donor 5 under HD and MD. Nevertheless, the small pellets from the LD for both donors 4 and 5 were also positively stained. Donor 6 was characterized by very light staining in all three concentrations (μ-bar: 500 μm). After the repeated sections, the pellet from donor 1 was completely cut through, and no additional staining could be performed.

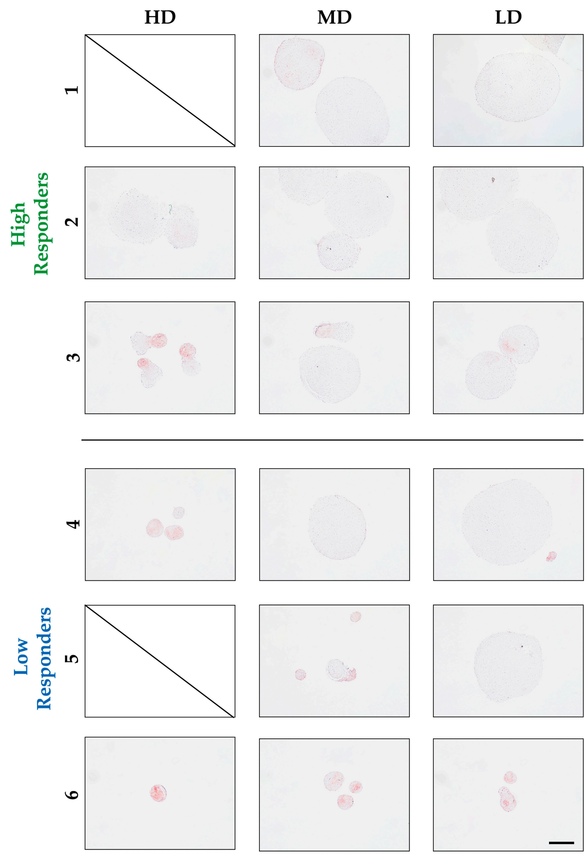

Figure S7. Matrix Metalloproteinase-3 (MMP-3) histological staining under the different BSA concentrations to further characterize the hypertrophic and osteogenic differentiation of the pellets. Overall, the high responders were negative under MD and LD, with some slight increase of the staining on the edges for donor 1 and central areas of MD for donor 3, which was however positive under HD. From the low responders, donors 4 and 5 were, in general negative, under MD and LD, with some of the adjacent small pellets positive in MD for donor 5, and the small pellets of donor 4 under HD. Donor 6, however, had the highest intensity, particularly on the pellet under HD. (μ-bar: 500 μm). The small pellets from donors 1 and 5 at HD were completely sectioned, hindering their visualization for this staining procedure.
